# How to write a medical CV

**DOI:** 10.1097/IJ9.0000000000000032

**Published:** 2017-06-15

**Authors:** Riaz Agha, Katharine Whitehurst, Daniyal Jafree, Yadsan Devabalan, Kiron Koshy, Buket Gundogan

**Affiliations:** a Guy’s and St Thomas’ NHS Foundation Trust; b Royal Devon and Exeter Hospital; c University College London Medical School; d UCL Medical School, University College London; e Brighton and Sussex University Hospitals; f University College London, UK

**Keywords:** curriculum vitae, CV, surgical training, portfolio, audit

## Abstract

A medical curriculum vitae remains an important document that has 2 main roles: to distinguish candidates applying for various positions, whether that be jobs, posts, grants and it provides a means of keeping an up-to-date record of all your achievements and skills gained thus far. This article provides detailed guidance on how to structure an effective curriculum vitae to maximize your chances of success when applying for these positions.

A curriculum vitae (CV) serves as an important means of helping you to secure an interview or a job. Although many medical job applications such as the foundation program are submitted online without the need for a CV, it still remains important when applying to surgical training, any consultant posts, general practice training, portfolio assessments, electives, and grants^1^,^2^. It also functions as a way of recording all your achievements, skills, and experiences and keeping them up to date. This will help to identify areas which still need improving or which areas are particularly relevant for the role you are applying to. Therefore, it is important to review your CV regularly for each new role or purpose that you are using it for and tailor it accordingly^3^.

## Length

There is no required length for medical CVs, with the general trend to be 2–3 pages of A4 that keeps the information succinct and relevant. An academic CV may tend to be longer, with research and publications being included.

## Top tips

- Before starting to write the CV, identify exactly what the role/person specification is looking for and ensure that they will be able to identify straightaway from your CV that you are applying for that role.
- A CV has to make an immediate impact, so good presentation is vital—nice paper quality, clear font 12-point Arial or Times, clear layout with enough white space margins, avoid large chunks of text, use bullet points, ensure no spelling mistakes.
- Style of writing—professional short and simple sentences, use active words when referring to skills, focus on positive aspects.
- Do not exaggerate or fabricate any information on the CV. Also do not include everything you have done if it is not relevant to the application. There will be plenty of opportunity at interviews to get this information across if it is important.

The following headings are useful to ensure a good structure to your CV.

## Cover letter

The first page is usually independent and just contains what role you are applying for, a brief introduction to you and why you want to apply for this role. It serves more of an administrative purpose.

## Personal details

This should include your full name and abbreviated qualifications (eg, MBBS, BSc). Contact details including address, telephone number, and email address should be provided. Ensure that these are professional contact details and not personal ones. Other details such as date of birth, nationality, and sex are optional and should not make a difference to your application. It is also a good idea to include general medical council number and medical defense numbers once you have been registered.

## Career statement

This is a useful and quick way to highlight how you are suitable for the role in question by stating your most relevant experience and skills as well as express your professional goals regarding your future career^4^.

## Education and qualifications

This should list the most recent qualification first. For university degrees, include details of any special study modules or electives if they are relevant to the role. Qualifications gained in school such as GCSEs and A-levels can be stated with a summary line that includes the institution, year, and grades.

## Present position

Highlight your current role and what responsibilities and skills it entails, including any relevant information such as the hospital, supervisor name, and date started.

## Career history

Similar to the previous section, include a brief summary of the job description and any other relevant information especially the dates. Order this with the most recent job first. There is no need to include every single job that you have ever had especially if it is not relevant.

This section can be expanded upon with a “clinical skills” section where you can expand on any relevant experiences and skills that are relevant to the new role, for example highlighting any surgical skills gained that are needed for a new surgical job.

## Voluntary/work experience

This is a good section to include any experience gained either medically, such as any specialty experience taster days. Non-medical experience such as charity work and any volunteering are also useful. For both it is useful to highlight what you have gained from it.

## Audit and quality improvement work

Participating in clinical audits or quality improvement works is important in a medical career and will provide points in speciality applications^5^. Therefore, you should clearly show any audits that you have been involved in including the dates, topic, your role, any guidelines used, your conclusions, and future outcomes.

## Management and leadership

Management and leadership skills are vital for a doctor, and this can be highlighted through clear examples either medically or nonmedically related^6^. Examples include positions in committees, supervising juniors, and organizing events^7^.

## Prizes and awards

This can include awards received both in education and at work. Again, list the most recent one first and highlight the ones that are most relevant to the role in question.

## Publications and presentations

This is important in academic CVs in particular, but is also relevant in any medical CV. State your publications in the same format that it would appear in a journal and include a pubmed ID number if available. This section also includes any posters or presentations that you have produced from any research projects or audits.

## Teaching experience

This is another skill that is vital for doctors, so include any teaching experience either formal or informal at any level, the topics and audience taught and what you gained from it. Have some feedback forms within your portfolio as evidence of this.

## Training courses and educational symposia

Include training courses and symposiums that add value to your CV, for example a suturing course will look good for surgical applications, as will basic and advanced life support courses. Courses related to examination preparation are usually not relevant. The courses can also relate to other aspects such as management, teaching, or research. For each one state the date and title of the course, and can be presented either chronologically or in order of importance. Importance can further be split up into international, national, or regional courses.

## Information technology skills

Many workplaces now require competent information technology skills such as prescribing drugs in hospital, so make sure to include any relevant experience and skills here^8^. Examples can include basic software such as the Microsoft packages but also any specialist software such as statistics packages, databases for research.

## Professional and society memberships

This usually applies to ones where you have been elected toward, as opposed to ones you pay a subscription fee to join.

## Personal interests

This is designed to present you as a well-balanced individual and to catch the reader’s attention. Include any extracurricular activities that you do but rather than listing them, demonstrate why you do them and what you have gained from them especially how they have improved your ability as a doctor. It is important not to fabricate any information this section as you can be quizzed about it during an interview.

## Referees

You should list at least 2 references, either with their name and contact details present or state “available upon request.” However, it is a good idea to ensure that your referees have agreed to act as such and they are aware of what role you are applying for so they can tailor their reference accordingly^9^.
